# Long-Term Treatment with Citicoline Prevents Cognitive Decline and Predicts a Better Quality of Life after a First Ischemic Stroke

**DOI:** 10.3390/ijms17030390

**Published:** 2016-03-16

**Authors:** Jose Alvarez-Sabín, Estevo Santamarina, Olga Maisterra, Carlos Jacas, Carlos Molina, Manuel Quintana

**Affiliations:** 1 Department of Neurology, Hospital Vall d’Hebron, Universitat Autónoma de Barcelona, Barcelona 08035, Spain; esantama@vhebron.net (E.S.); omaisterra@vhebron.net (O.M.); cmolina@vhebron.net (C.M.); maquinta@vhebron.net (M.Q.); 2 Department of Psychiatry, Hospital Vall d’Hebron, Universitat Autónoma de Barcelona, Barcelona 08035, Spain; cjacas@vhebron.net

**Keywords:** quality of life, ischemic stroke, citicoline, cognitive impairment

## Abstract

Stroke, as the leading cause of physical disability and cognitive impairment, has a very significant impact on patients’ quality of life (QoL). The objective of this study is to know the effect of citicoline treatment in Qol and cognitive performance in the long-term in patients with a first ischemic stroke. This is an open-label, randomized, parallel study of citicoline *vs.* usual treatment. All subjects were selected 6 weeks after suffering a first ischemic stroke and randomized into parallel arms. Neuropsychological evaluation was performed at 1 month, 6 months, 1 year and 2 years after stroke, and QoL was measured using the EuroQoL-5D questionnaire at 2 years. 163 patients were followed during 2 years. The mean age was 67.5 years-old, and 50.9% were women. Age and absence of citicoline treatment were independent predictors of both utility and poor quality of life. Patients with cognitive impairment had a poorer QoL at 2 years (0.55 *vs.* 0.66 in utility, *p* = 0.015). Citicoline treatment improved significantly cognitive status during follow-up (*p* = 0.005). In conclusion, treatment with long-term citicoline is associated with a better QoL and improves cognitive status 2 years after a first ischemic stroke.

## 1. Introduction

Although age-standardized rates of stroke mortality have decreased worldwide in the past two decades, the absolute number of people who have a stroke every year, stroke survivors, related deaths, and the overall global burden of stroke (DALYs lost) are great and increasing [1]. We must remain mindful that substantial post-stroke disability is considered a worse outcome than death for many people. The presence of stroke represents a nearly 1.9 fold increase in cognitive decline [2]. Almost half of stroke survivors have cognitive impairment [3]. Cognitive decline after stroke is even more common than stroke recurrence [4]. Cognitive impairment causes loss on independence for activities of daily living and may accelerate the need of institutionalization [5,6], and quality of life declines after first ischemic stroke [7]. Thus, an increased focus on stroke survivors, in particular of their cognitive and physical function, is needed. Quality of life (QoL) after stroke is of vital concern for survivors, so understanding the factors that can influence on it is necessary to help stroke survivors.

Among various scales available for measuring QoL, one of the most commonly used is the EuroQoL-5D, a scale that has demonstrated its value and reliability for measuring quality of life in patients who have suffered a stroke [8,9].

Our objective is to know the effect of citicoline treatment in Qol and cognitive performance in the long-term in patients with a first ischemic stroke.

## 2. Results

**Figure 1:**
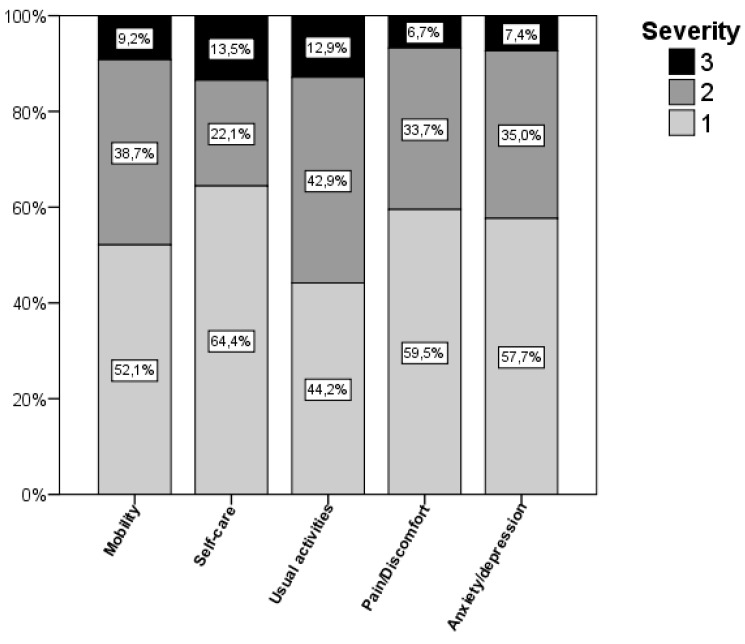
Quality of life according to the level of each EuroQol-5D dimension. Usual activities was affected in a higher number of patients.

A total of 163 patients who had suffered a first ischaemic stroke were assessed after 2 years. The mean age was 67.5 ± 10.7 years and 83 (50.9%) were women. Among the risk factors, 60.1% of patients had hypertension, 40.5% dyslipidaemia and 30.9% diabetes mellitus; 29.4% were smokers, and 12.3% had atrial fibrillation. Stroke severity, measured using baseline NIHSS, was 13 (10–16). 86 patients (52.8%) were being treated with citicoline and 77 (47.2%) were not. Baseline characteristics of patients that received and did not received citicoline are shown in Table 1. During the follow-up period, 13 patients (8%) had stroke recurrence, 7 (8.1%) treated with citicoline and 6 (7.8%) not treated (*p* = 0.935). Quality of life of these patients at 2-year follow-up according to the EuroQoL-5D questionnaire is shown in Figure 1, where the involvement of each dimension on the scale is represented. The mean overall Utility index was 0.63 ± 0.28, with 37 (22.7%) patients being classified within the group with poor or very poor quality of life (utility < 0.5). The median VAS scale score was 70 (50–85) with a range from 0 to 100.

The comparison of utility with demographic variables, risk factors and severity (Table 2) showed that women who had suffered a stroke had a worse quality of life (0.58 *vs.* 0.67; *p* = 0.030). Similarly, older patients (*p* = 0.046), with a higher initial stroke severity (*p* = 0.016) and those with PAD (*p* = 0.047) were also significantly correlated with a worse utility. Patients with previous hypertension presented a tendency to have a worse quality of life (0.59 *vs.* 0.67; *p* = 0.078), although this difference was not statistically significant. The analysis between patients who took citicoline and those who did not, showed that the patients being treated with this drug had significantly better QoL than the rest (0.67 ± 0.26 *vs.* 0.58 ± 0.30; *p* = 0.041). A multivariate analysis using a linear regression model showed age (β =−0.005 [95%CI: −0.009, −0.001]; *p* = 0.024) and treatment with citicoline (β = 0.112 [95%CI: 0.027, 0.198]; *p* = 0.010) as independent predictors of utility. Thus, for each 10-year increase in patient’s age, utility decreased by a mean of 0.05 points; therefore quality of life worsens with age. Furthermore, irrespective of age, patients who took citicoline improved their quality of life according to this index by a mean of 0.112 points.

**Figure 2:**
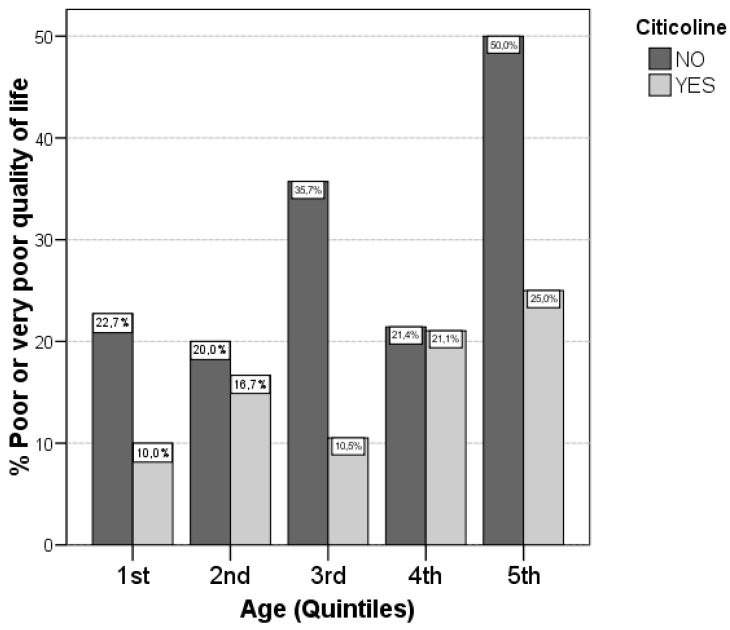
Citicoline compared with quality of life for different age groups. Patients not treated with citicoline were more likely to have a poor quality of life in all age subgroups. Quintiles: 1st: <60, 2nd: 60–64, 3rd:65–70, 4th:70–75, 5th: >75.

The variables associated with poor or very poor quality of life are shown in Table 3. Only old age was significantly associated with a poor quality of life (70.7 *vs.* 66.6; *p* = 0.039). Moreover, although not achieving statistically significant differences, there was a trend for females (*p* = 0.054), patients with hypertension (*p* = 0.069), and those with PAD (*p* = 0.079) of having worse quality of life. When treatment with citicoline was analysed, we observed that 28.6% of patients not receiving this drug had a poor or very poor quality of life compared with 17.4% of those under treatment, these differences did not achieve statistical significance (*p* = 0.090). After performing a logistic regression analysis, we observed that age (OR 1.048 [95%CI: 1.010, 1.087]; *p* = 0.012) and the absence of citicoline treatment (OR 2.321 [95%CI: 1.057, 5.100]; *p* = 0.036) were independent predictors of poor or very poor quality of life. After adjusting for age, treatment without citicoline was associated with a worse quality of life. As seen in Figure 2, treatment with citicoline is associated with a better quality of life, both in older age groups as well as in younger patients.

In the VAS assessment, no significant differences were observed, but some variables as age (*p* = 0.073), sex (*p* = 0.051) and PAD (*p* = 0.074) tended to be associated in the same way as they were with the utility. Patients treated with citicoline had also better scores on this scale (72.5 (50–90) *vs.* 70 (40–80), *p* = 0.063), although differences did not achieve statistical significance.

**Figure 3:**
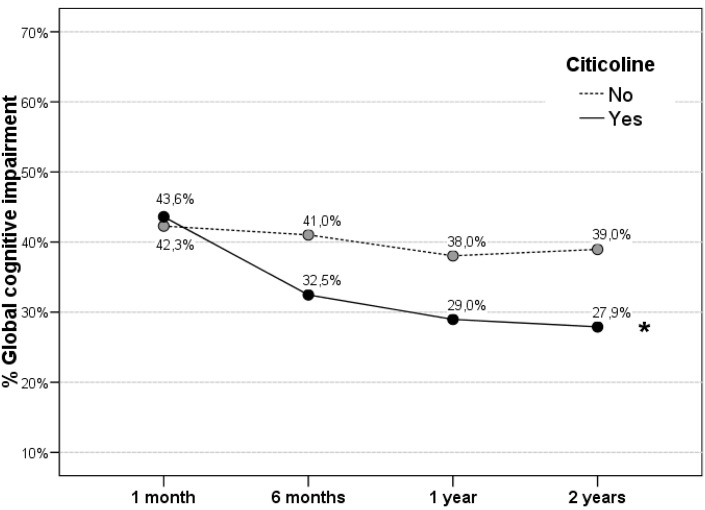
Global cognitive impairment during follow-up. Patients treated with citicoline show a significant improvement in cognitive status during follow-up (* *p* = 0.005). After the first year, only citicoline-treated patients continue to improve cognitive status.

Patients treated with citicoline had less cognitive impairment at 2 year follow-up (27.9% *vs.* 39%) without achieving statistically significant differences. However, citicoline group showed a significant improvement during follow-up (*p* = 0.005). (Figure 3). Untreated group did not show significant changes.

**Figure 4:**
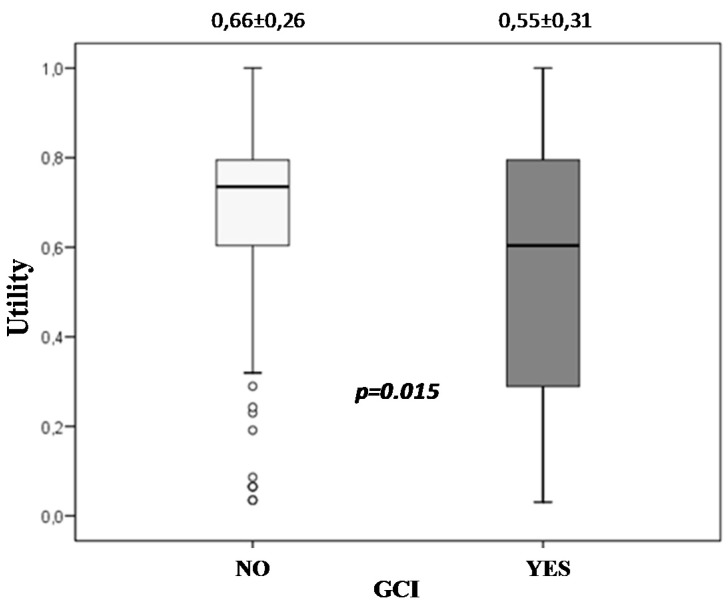
Global cognitive impairment and quality of life. Patients with GCI had a poorer quality of life at 2 years post ischemic stroke.

Moreover, GCI was associated with a poorer quality of life at 2 years (0.55 *vs.* 0.66 in utility, *p* = 0.015). (Figure 4).

## 3. Discussion

As the leading cause of cognitive and physical disability, stroke has a very significant impact on the quality of life of patients. The mean utility index in our study is 0.63 ± 0.28 and nearly one quarter (22.7%) of the patients present a poor or very poor quality of life two years after a first ischaemic stroke. Treatment with long-term citicoline improves cognitive status of stroke patients and is associated with a better quality of life at 2 years.

In previous studies where post-stroke QoL was measured [10,11,12,13,14,15,16,17,18,19,20,21], the utility measurement varied between 0.47 and 0.88 (Table 4), which could be explained by the fact that the follow-up times varied greatly between studies (from 3 months to 5 years) and that the type of stroke evaluated was different from one study to another. These publications showed that patients with a better QoL, *i.e.*, an utility of 0.88, are TIA patients with 3 months of follow-up [19], and conversely those patients with a worse QoL, *i.e.*, an utility of 0.47, are individuals with longer follow-up (2 years) including ischemic and hemorrhagic strokes [10,15]. Although stroke type clearly had an influence, the follow-up time did not seem to be as important in measuring quality of life, since according to the studies by Luengo-Fernandez R., *et al.* [17] and López-Bastida J., *et al.* [15], the values of this index did not change significantly between 6 months and 5 years of follow-up. Only an American study of first ischaemic strokes [7] found a significant worsening in quality of life starting in the 3rd year, although the quality of life index was measured in a different way (QLI) and there were no significant changes in those patients with private health insurance.

The mean utility in our study was similar to two previous publications in which quality of life in ischaemic strokes was evaluated [11,17]; 0.68 at the 4-year follow-up in the German study [11] and 0.66 at the 2-year follow-up in the OXVASC trial from the UK [17]. The VAS scale values in the trials evaluating stroke patients were similar to ours, with mean scores ranging from 51.6 to 67.3 (Table 4). Taking into account each of the dimensions of the EuroQoL-5D scale, we observed that usual activities were the most disrupted in our study, affecting more than half of the patients. This was in correlation with data of most of previous studies [13,18,20,22,23], having also patients more affected in usual activities.

In the current study, women had worse quality of life at 2 years compared to men, which agrees with the results of other studies [10,11,12,17,19,21]. If we focus on studies evaluating only ischaemic stroke, men were significantly better in the OXVASC study [17] and it was even described as an independent predictor of good quality life in a multivariate analysis; similar results were obtained in the American study by Bushnell, C.D., *et al.* [21]. The German study [11] did not show statistically significant differences although women had less quality of life. Furthermore, there is a more recent study including only patients with ischaemic stroke (BASIC Project) [24] in which again a worse prognosis was also found in the quality of life of women, being highly statistically significant after an adjusted regression model. Similarly to most studies [7,10,11,12,17,19,25], older age was correlated with a worse quality of life in our patients, remaining as an independent predictor in a multivariate analysis, in agreement with most studies [7,10,17,19,22,25]. In our study, we also found a statistically significant correlation with the initial stroke severity. This correlation was also established in all the articles that evaluated this issue [7,10,13,17,19,21,23], and it was reported as an independent predictor in most of them [7,10,17,19,21,23]. In this study, we found that treatment with citicoline was an independent predictor of utility with a beta of 0.112 in the regression model. This means that the mean utility of patients receiving citicoline is 0.112 higher than the rest of patients. Although this difference is statistically significant, it does not seem to be a substantial change. However, it is difficult to establish if this difference is clinically relevant or not, since there is not any known cutoff validated in stroke patients.

Demonstrated efficacy of citicoline in improving cognitive functions after an ischaemic stroke [26] may be responsible for this beneficial effect on post-stroke quality of life. Three studies in the literature measured patient cognitive status using the mini mental examination [11,18,22] and in all of them there were highly significant correlations with the utility, which would reinforce our hypothesis that long-term treatment with citicoline at high doses would be associated with a better quality of life of patients by improving patients’ neurocognitive function after a first ischemic stroke. Our study also shows a progressive and significant improvement in cognitive status during follow-up when patients are treated with long-term citicoline. This improvement is maintained even after the first year post stroke, a period from which untreated patients begin to show a slight decrease of cognitive functions, which could be an age-dependent effect. Moreover, the results of the present study also show that patients with GCI have a poorer quality of life.

Apart from its neuroprotective effects, citicoline also possesses a substantial neuroregenerative potential [27,28,29,30,31,32,33,34] that may explain better its beneficial effects in post-stroke cognitive impairment and quality of life. Since experimental studies have shown that neurorepair mechanisms initiated after cerebral ischemia remain active beyond twelve months [35], the use of drugs that enhance neurorepair mechanisms as citicoline [27,28,29,30,31,32,33,34], would be indicated for periods of time longer than one year in order to improve post-stroke sequelae and prevent cognitive decline associated with age, as shown by the results of this study.

The study has some limitations. One of them is that the open-label design of the study could have influenced our results; more specifically, some cognitive improvement in the citicoline group might be attributable to patients’ expectation bias, so placebo-controlled studies are needed to better elucidate the efficacy of citicoline in stroke patients. Another limitation of this study is that only the evaluation of the quality of life at 2 years was contemplated. Having evaluations during early time points would have probably allowed us having an idea of critical timing in improvement or drug administration. However, considering the changes in cognitive performance and the relationship between cognitive impairment and quality of life, it makes us think that quality of life of patients could have a similar evolution in the follow-up.

In conclusion, stroke negatively impacts quality of life, especially in usual activities. Nearly one quarter of ischaemic stroke survivors have a poor or very poor quality of life 2 years after the stroke. Citicoline independently predicts a better quality of life at 2 years and improves cognitive status of stroke patients during follow-up.

## 4. Materials and Methods

### 4.1. Patients

The patients participated in a study designed to measure the effect of long-term treatment with citicoline (1g/day orally) on the cognitive functions of patients who had suffered a first ischaemic stroke [26]. Appropriated secondary stroke prevention measures were used in all recruited patients. When indicated, neurorehabilitation treatment was made according to the Stroke Unit protocol of the Vall d’Hebron Hospital.Global cognitive status was defined as the mean of all standardized (0–100) cognitive functions scores. It was considered as global cognitive impairment (GCI) an overall score <40. More details on the neuropsychological assessment are described in the previous study of Alvarez-Sabín J, *et al.* [26]. For the present study, we evaluated the patients who had completed the 2-year follow-up. During all the follow-up time no patient received any drug that could modify cognitive functions or alertness.

The study was approved by Vall d’Hebron Hospital IRB; informed consent was obtained in all patients.

### 4.2. Quality of Life

Patient quality of life was measured using the EuroQoL-5D health questionnaire adapted for use in Spain [36], in which five health dimensions were assessed: mobility, self-care, usual activities, pain/discomfort and anxiety/depression, each with three levels of severity (1: no problems, 2: some problems or moderate problems, 3: serious problems). Based on this scale we were able to obtain an overall index [37] (utility) ranging from 0 to 1, where 1 represented the patient without any health problems or with the best possible health status and 0 the worst health status or death. Starting from the best possible health status (utility = 1), each dimension affected could be penalised differently on this scale [37]. This index was distributed similarly to the study of Christensen et al. [38], where utility distribution revealed two groups of patients according to a cut-off point of 0.5. Thus, in line with other studies [15,19], a utility score <0.5 was used to identify those patients with a poor or very poor quality of life. These patients were characterised by being affected in some way in all dimensions or by having a level 3 severity in at least one of them. The visual analogue scale (VAS) of the EuroQol-5D questionnaire was also assessed. This scale measures the patient’s subjective state using a thermometer with scores ranging from 0 to 100, where 0 is the worst imaginable health status and 100 the best imaginable health status.

### 4.3. Statistical Analysis

Statistical analysis was performed using the SPSS Statistics 17.0 software (SPSS Inc., Chicago, IL, USA).

The comparisons made with the utility index were assessed using Student’s *t*-test for categorical variables and Pearson’s correlation coefficient for continuous variables, except for the NIHSS scale which was assessed with the Spearman’s correlation coefficient. A multiple linear regression analysis was performed to obtain variables independently associated with a worse utility, including in the model those variables with significance under 0.1 in the bivariate analysis.

The variables related with GCI and a worse quality of life (utility < 0.5) were assessed using Pearson’s Chi-square test or, if necessary, Fisher’s exact test in categorical variables, and Student’s *t*-test in numeric variables, except for NIHSS where the Mann-Whitney *U* test was applied. The variables independently associated with a poor or very poor quality of life were obtained with a logistic regression model using the forward selection stepwise method, including variables with significance under 0.1 in the bivariate analysis.

The comparisons made with the VAS were evaluated using Spearman’s correlation coefficient in continuous variables and the Mann-Whitney *U* test in categorical variables. Changes in GCI during follow-up were assessed using the linear trend chi-square test.

*p*-Values < 0.05 were considered statistically significant in all comparisons.

## Figures and Tables

**Table 1:** Baseline characteristics in patients who received and did not received citicoline.

Parameters	Citicoline		*p*
	Yes (*n* = 86)	No (*n* = 77)	
Sex (female)	54.7%	46.8%	0.314
Age	68.5 ± 9.8	66.4 ± 11.4	0.200
Years of education	5.8 ± 3.7	5.1 ± 3.3	0.173
Smoking	24.4%	35.1%	0.137
Alcohol	17.4%	24.7%	0.256
Dyslipidaemia	34.9%	46.8%	0.123
Diabetes mellitus	38.4%	24.7%	0.061
Hypertension	59.3%	61%)	0.821
Atrial Fib.	12.8%	11.7%	0.830
Coronary heart disease	4.7%	10.4%	0.161
AMI	11.6%	14.3%	0.613
PAD	4.7%	5.2%	1.000
Baseline NIHSS	13 (10–16)	14 (10–16.5)	0.518

**Table 2:** Utility according to demographic variables, risk factors, and severity.

Parameters		Utility	*p*
Sex	Male	0.67 ± 0.27	0.030
	Female	0.58 ± 0.29	
Age		R: −0.156	0.046
Years of education		R: 0.081	0.306
Smoking	No	0.62 ± 0.27	0.670
	Yes	0.64 ± 0.31	
Alcohol	No	0.64 ± 0.29	0.306
	Yes	0.58 ± 0.27	
Dyslipidaemia	No	0.63 ± 0.26	0.763
	Yes	0.62 ± 0.31	
Diabetes mellitus	No	0.64 ± 0.28	0.316
	Yes	0.59 ± 0.29	
Hypertension	No	0.67 ± 0.26	0.078
	Yes	0.59 ± 0.29	
Atrial Fib.	No	0.64 ± 0.27	0.199
	Yes	0.53 ± 0.35	
Coronaryheart disease	No	0.62 ± 0.29	0.521
	Yes	0.68 ± 0.24	
AMI	No	0.62 ± 0.28	0.465
	Yes	0.67 ± 0.30	
PAD	No	0.64 ± 0.28	0.047
	Yes	0.43 ± 0.30	
Baseline NIHSS		R: −0.188	0.016

R: correlation coefficient.

**Table 3:** Variables associated with poor or very poor quality of life.

Parameters	Quality of Life		*p*
	Good/Acceptable	Poor/Very Poor	
Sex (female)	46.8%	64.9%	0.054
Age	66.6 ± 10.2	70.7 ± 12.1	0.039
Years of education	5.7 ± 3.6	4.8 ± 3.2	0.152
Smoking	29.4%	29.7%	0.966
Alcohol	19.8%	24.3%	0.555
Dyslipidaemia	40.5%	40.5%	0.994
Diabetes mellitus	31.0%	35.1%	0.631
Hypertension	56.3%	73.0%	0.069
Atrial Fib.	10.3%	18.9%	0.161
Coronary heart disease	7.9%	5.4%	0.604
AMI	13.5%	10.8%	0.669
PAD	3.2%	10.8%	0.079
Baseline NIHSS	13 (10–16)	14 (11–17)	0.301

**Table 4:** Literature review of recent quality of life studies using the EuroQoL-5D scale in stroke.

Study	Year	Country	Follow-up	*n*	Stroke Type	Utility or Equivalent	VAS	Sex	Age	NIHSS	Multivariate (Worse Quality of Life)	Other
Sturm, J.W., *et al.* [10]	2004	Australia	2 years	225	Ischaemic and haemorrhagic	0.47 (95% CI 0.42–0.52)	N.A.	Females worse (sig.)	Old age worse (sig.)	Worse with higher NIHSS (sig.)	Age, sex, NIHSS, and socioecon. status	–
Haacke, C., *et al.* [11]	2006	Germany	4 years	54	Ischaemic	0.68 ± 0.33	56.5	Females worse (n.s.)	Old age worse (sig.)	N.A.	IB, anal continence, continence and depression.	Worse with lower IB, higher mRS, impairment (MMSE) and depression.
Xie, J., *et al.* [12]	2006	USA	>1 year	1040	Stroke	0.69 (SE 0.01)	61.6 (SE 0.08)	Females worse	Worse in old age	N.A.	N.A.	–
Pinto, E.B., *et al.* [13]	2011	Brazil	2 years	67	Stroke	0.52 ± 0.36	N.A.	N.A.	No correlation with age (n.s.)	Worse with higher NIHSS (sig.)	N.A.	–
Hansson, E.E., *et al.* [14]	2012	Sweden	1 year	283	Stroke	0.5 ± 0.39	62.5 ± 21.8	N.A.	N.A.	N.A.	N.A.	–
López-Bastida, J., *et al.* [15]	2012	Canary Islands, Spain	1 year	94	Stroke	0.49 ± 0.42	56 ± 27	N.A.	N.A.	N.A.	N.A.	Quality of life does not change in 1–2–3 years.
			2 years	205		0.47 ± 0.44	51.6 ± 27					
			3 years	149		0.46 ± 0.45	55 ± 25					
Hornslien, A.G., *et al.* [16] (SCAST)	2012	Northern Europe	6 months	870	Stroke: Candesartan Placebo	0.74 (0.59–0.88) 0.78 (0.62–0.88)	66 ± 20	N.A.	N.A.	N.A.	N.A.	MMSE: 28 (25–29); Does not compare it with quality of life.
				882			67.3 ± 19					
Luengo-Fernández, R., *et al.* [17] (OXVASC)	2013	UK	1 month	314	Ischaemic stroke	0.64 ± 0.33	N.A.	Females worse (sig.)	Old age worse (sig.)	Worse with higher NIHSS (sig.)	Sex, Age, NIHSS, risk factors, stroke type	Does not vary in 1–5 years.
			1 year			0.70 ± 0.27						
			2 years			0.66 ± 0.29						
			5 years			0.67 ± 0.31						
Sprigg, N., *et al.* [18] (ENOS)	2013	Countries worldwide	3 months	2238	Ischaemic and haemorrhagic	N.A.	65.8 ± 22.4	N.A.	N.A.	N.A.	N.A.	Worse with lower IB, higher mRS, impairment (MMSE) and depression.
Wang, Y.-L., *et al*. [19] (CHANCE)	2014	China	3 months	5104	TIAs	0.88 ± 0.21	84 ± 15	Females worse (sig.)	Old age worse (sig.)	Worse with higher NIHSS (sig.)	Age, hypertension, DM, NIHSS, and various treatments	Worse at higher mRS.
							89 (80–85)					
Golicki, D., *et al.* [20]	2014	Poland	4 months	112	Stroke	0.691 ± 0.267	60.7 ± 22.4	N.A.	N.A.	N.A.	N.A.	Correlation with Barthel and mRS
							60 (45.5–80)					
Bushnell, C.D., *et al.* [21]	2014	USA	1 year	1370	Ischaemic (including TIAs)	0.83 (0.74–1)	N.A.	Females worse (sig.)	N.A.	Worse with higher NIHSS (sig.)	NIHSS and sex	No changes in quality of life during 1 year
Current study	2015	Spain	2 years	163	First ischaemic stroke	0.63 ± 0.28	64.4 ± 25 70 (50–85)	Females worse (sig.)	Old age worse (sig.)	Worse with higher NIHSS (sig.)	Age, treatment with citicoline	–
						0.70 (0.59–0.79)						

N.A.: Not Available; n.s.: not significant; sig.: statistically significant; CI: Confidence interval; SE: Standard error; MMSE: Mini Mental State Examination.

## Abbreviations

QoL	Quality of Life
VAS	Visual Analogue Scale
NIHSS	National Institutes of Health Stroke Scale
TIA	Transient Ischemic Attack
PAD	Peripheral Arterial Disease
GCI	Global Cognitive Impairment
